# Application of remote sensing to understand the role of Galician feral horses in the biomass reduction of a shrub-grassland-dominated landscape

**DOI:** 10.1186/s12862-024-02276-5

**Published:** 2024-07-02

**Authors:** Andrea Janeiro-Otero, Xana Álvarez, Carsten F. Dormann

**Affiliations:** 1 Department of Biometry and Environmental System Analysis, Faculty of Environment and Natural Resources, University of Freiburg, Tennenbacher Straße 4, 79106 Freiburg, Germany; 2 School of Forestry Engineering, University of Vigo, Campus A Xunqueira s/n, 36005 Pontevedra, Spain

**Keywords:** Wildfires, Galician feral horses, Livestock grazing, Drones, Fire behaviour, Fire management; forest management

## Abstract

Galician forests in northwestern Spain are subject to frequent wildfires with high environmental and economic costs. In addition, due to the consequences of climate change, these fires are becoming more virulent, occurring throughout the year, and taking place in populated areas, in some cases involving the loss of human life. Therefore, forest fire prevention is even more relevant than mitigating its consequences. Given the costs involved in forestry work, alternative measures to reduce fuel load and create vegetation gaps are needed. One involves grazing by an endemic species of feral horses (*Equus ferus atlanticus*) that feed on thicket-forming gorse (*Ulex europaeus*). In a 100-ha forest fenced study area stocked with 11 horses, four 50 m^2^ enclosed plots prevented the access of these wild animals to the vegetation, with the aim of manipulating their impact on the reduction of forest biomass. The measurement of biomass volumes is an important method that can describe the assessment of wildfire risks, unfortunately, high-resolution data collection at the regional scale is very time-consuming. The best result can be using drones (unmanned aerial vehicles - UAVs) as a method of collecting remotely sensed data at low cost. From September 2018 to November 2020, we collected information about aboveground biomass from these four enclosed plots and their surrounding areas available for horses to forage, via UAV. These data, together with environmental variables from the study site, were used as input for a fire model to assess the differences in the surface rate of spread (SROS) among grazed and ungrazed areas. Our results indicated a consistent but small reduction in the SROS between 0.55 and 3.10 m/min in the ungrazed enclosured plots in comparison to their grazed surrounding areas (which have an SROS between 15 and 25 m/min). The research showed that radar remote sensing (UAV) can be used to map forest aboveground biomass, and emphasized the importance and role of feral horses in Galicia as a prevention tool against wildfires in gorse-dominated landscapes.

**Supplementary Information:**

The online version contains supplementary material available at 10.1186/s12862-024-02276-5.

## Introduction

As mega-fires and extreme wildfire seasons become more common [1, 2] and wildfires become more severe [3] with climate change, it is vital to apply treatments before wildfire suppression and postfire restoration are needed. Wildfire prevention must aim to both reduce the likelihood of a fire occurring and limit its spread if it does occur. The key management target is fuel load, i.e., vegetation biomass, which is critical to wildfire suppression [4]. Measures to reduce fire damage include, among others, an appropriate network of roads and water supplies, firebreaks and fire detection systems, and immediate and efficient intervention by ground crews. However, to prevent these fires from occurring first, fuel treatments should be executed in a timely manner [5, 6].

Fuel treatments primarily aim to disrupt the vertical and horizontal progression of fire (passage from surface fuels to ladder fuels to canopy fuels) as well as its horizontal progression, particularly from crown to crown [7, 8]. Activities aimed at reducing surface fuels (low vegetation, woody fuel, shrub layer) decrease the chances of surface fires igniting ladder fuels and canopy fuels [9]. Some of the main treatments used to modify forest fuels are pruning [10], thinning, fuel mastication [11], prescribed fire [9, 12] and livestock grazing [13–15].

Communities dominated by European gorse (*Ulex europaeus* L.*)* are considered one of the most fire-prone types of shrubland because of the high rate of fuel accumulation and flammability of the species [16]. Wildfires in such communities can produce high-intensity fires that may be very difficult to control by firefighting actions and thus pose a major threat to both human populations and forest resources. The abandonment of many rural areas in Europe and the higher incidence of forest fires have led to an increase in gorse biomass accumulation in some regions. Since vegetation is not used by animals or managed by humans, the spatial heterogeneity of natural landscapes increases, leading to more unpredictable fire patterns [17, 18].

Prescribed fires or herbicides are frequently perceived negatively by local communities [19], making the use of grazing animals a very acceptable and possibly effective method for controlling shrub encroachment and reducing the risk of fire through the elimination of dangerous fuel ladders. Additionally, grazing reduces the continuity of grass and shrub cover, thereby decreasing rapid fire propagation and preventing the transition to crown fires [9]. All of these practices can be categorized as “preventive silviculture”; their primary goal is to avoid fires by treating surface fuels and encouraging low-density and vertically discontinuous stands. This also helps to modify fire behaviour sufficiently so that some wildfires can be more easily extinguished [8].

In Spain, the peak of the fire season usually starts towards the end of May and extends for approximately 21 weeks. Only in 2022, more than 300,000 ha of land in Spain was destroyed [20], caused by more than 430 fires. This is especially relevant in fire-prone regions such as northwest Spain, where shrublands are an important part of the landscape, accounting for 20% of the total area and 30% of the forestland in the region [21].

The measurement of biomass volumes is an important method that can describe changes in the states and processes of ecosystems, including the assessment of wildfire risks. Unfortunately, high-resolution data collection at the regional scale is very time-consuming [22, 23]. Direct measurements in the field [24] followed by laboratory analyses, require intensive sampling campaigns, repeated across seasons and years, which often involve the destructive removal of plants [25].

Satellite imagery is increasingly being used to estimate aboveground biomass at continental and global scales over long time periods [26], but this approach has significant limitations, given that the best spatial resolution available from satellite remote sensing is approximately 60 cm (e.g., Quickbird) or 2 m in the case of open data such as Sentinel-2. Airborne LiDAR can use lasers to measure the sensor’s distance from the ground and the leaf canopy, producing accurate and fine spatial scale remote sensing estimates of vegetation biomass [27] but at a high cost [26] and rarely accounting for small branches and leaf canopy biomass [27, 28]. Terrestrial laser scanning (ground-based LiDAR) can be used to estimate biomass for individual trees [29, 30] but is time-consuming for stationary equipment, especially in remote areas and steep terrain.

Drones (unmanned aerial vehicles: UAVs) have recently gained prominence for collecting remotely sensed data at low cost [31]. UAV-based methods generate three-dimensional (3D) point clouds using structure from motion (SfM) techniques, which typically predict the volume of a solid object [32]. SfM techniques have primarily been developed for industrial applications such as precision agriculture [33], but they are also becoming increasingly useful for mapping natural vegetation communities [34]. The automation of data collection, processing, and analysis provides useful data for quantifying variations in biomass volumes. UAV-based remote sensing thus seems to be a promising approach for mapping vegetation biomass at local to regional scales.

In northwestern Spain (Galicia region) and Portugal, populations of the endangered Galician feral horse *Equus ferus atlanticus* [35, 36] graze freely. This endemic subspecies is a free-roaming animal considered to be a remnant of the feral horses that have lived in the Iberian Peninsula since the Pleistocene [37]. It is responsible for essential ecological processes such as the conservation of Atlantic heath (*Erica* spp.) priority habitats and the reduction of forest biomass (and thus of forest fires) through the consumption of gorses and heather bushes, as well as the creation of natural corridors that act as firebreakers. Gorse is a native hedge with waxy foliage that holds high amounts of oils that easily ignite and burn hot, making fire movement very rapid and difficult to control.

The purpose of this article is to use radar remote sensing (UAV) to map forest aboveground biomass, emphasizing the importance and role of feral horses in Galicia as a prevention tool against wildfires in gorse-dominated landscapes. We explored whether feral horses can help to mitigate the devastation caused by wildfires by consuming fuels through their specific grazing/browsing habits and thus reducing the horizontal and vertical continuity of fuels.

## Methods

### Study area

The present research was carried out in Fornelo de Montes. The area covers an extension of 1500 ha. Its average altitude is 712 m a.s.l., with a maximum of 1061 m a.s.l. The Atlantic climate experiences moderate temperature fluctuations (annual average temperature of 13 °C, monthly minimum temperature of 3 °C, maximum temperature of 23 °C) and abundant rainfall, with average wind speeds of 17 km/h.

Most of the area is covered by gorse, carqueja (*Genista tridentata)* and heather bushes (*Calluna vulgaris)*. Forests are limited to oak (*Quercus robur)* groves and riverside forests formed mainly by ash (*Fraxinus excelsior)* and birch (*Betula* spp.*)* trees in the lower areas and groups of planted red pine (*Pinus resinosa)* in the higher areas. There are commercial plots of eucalyptus (*Eucalyptus globules)* and pine (*Pinus sylvestris)* on sloping areas.

**Fig. 1:**
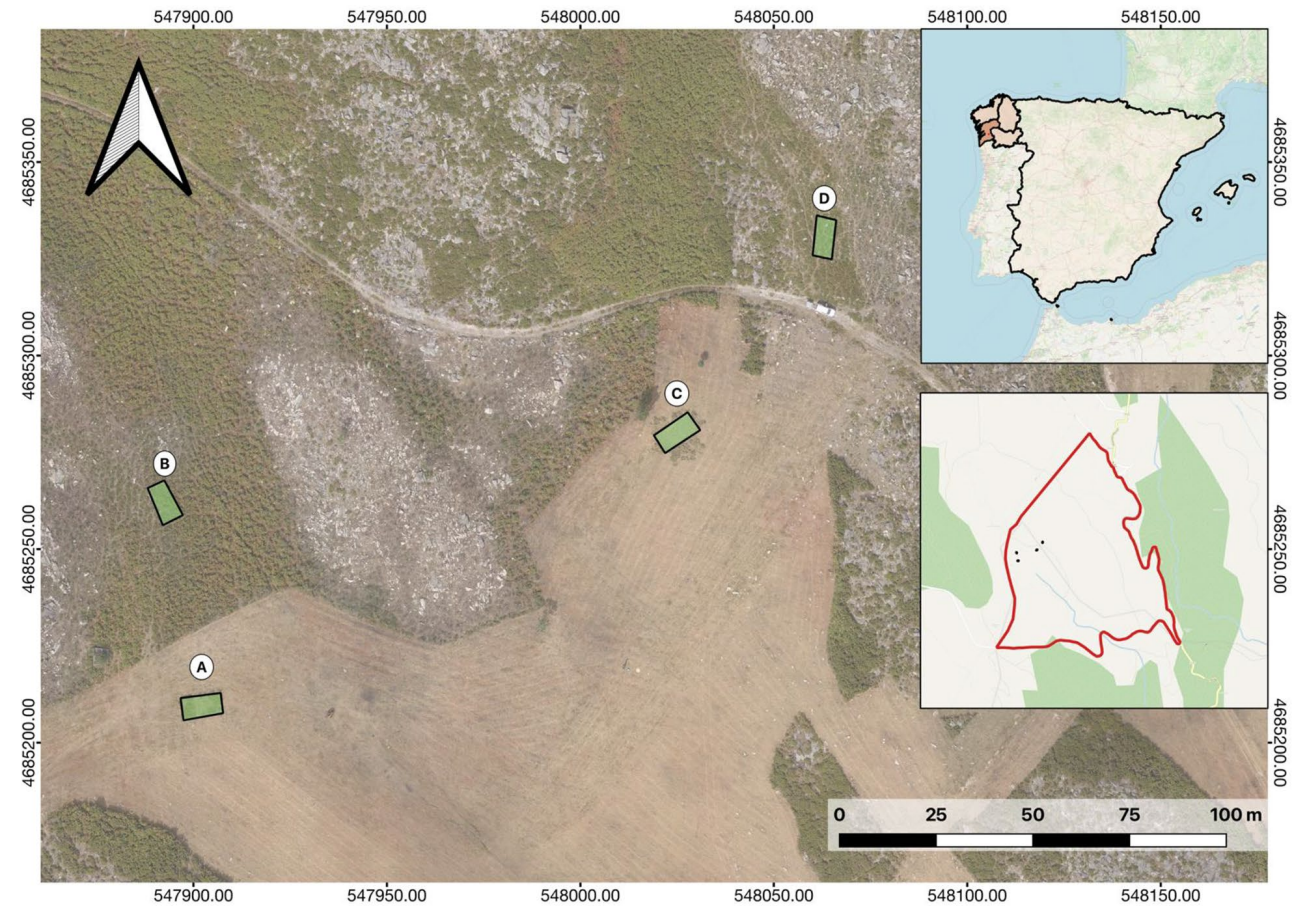
Map of the study area showing the four enclosed (**A-D**) plots inaccessible to Galician feral horses

**Fig. 2:**
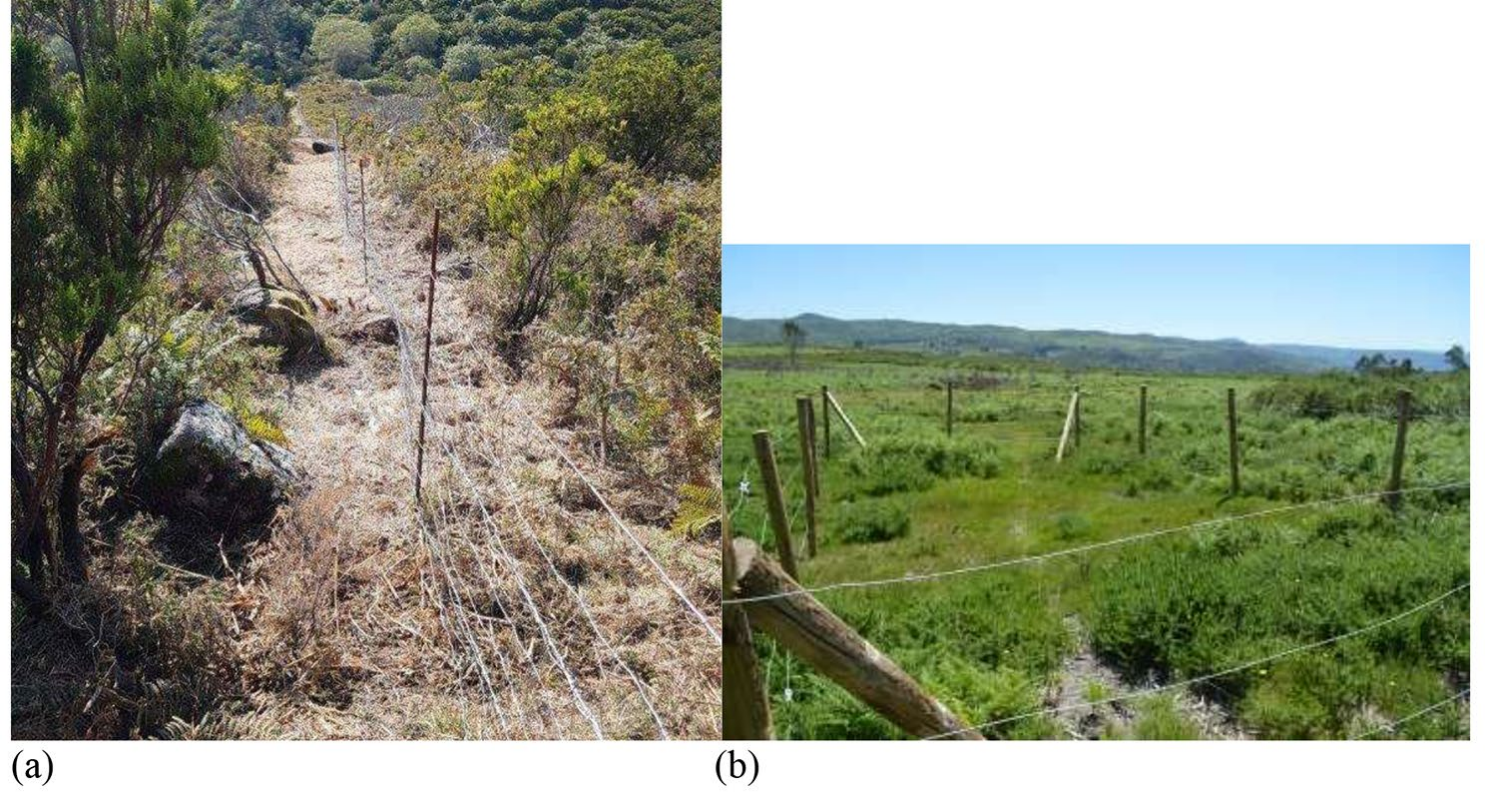
Fence surrounding the study area (**a**) and one of the enclosed plots that feral horses cannot access (**b**)

The study area (Fig. 1), with a total surface area of 100 ha, was enclosed around its perimeter with wooden posts and wire (Fig. 2); it offers a watering trough for cattle and Canadian steps that prevents animals from leaving the area. A total of eleven feral horses, eight mares, one male and two foals, were intentionally enclosed in the study area to assess their role in aboveground biomass reduction and, therefore, the prevention of wildfires.

To establish control plots where the animals had no influence and the vegetation grew naturally without any external constraints, four enclosures (named A-D) ranging from 54 to 59 m² were set up in different gorse areas (Fig. 1) combined with herbaceous plants of various ages within the study area, where the animals had no access. The location of these enclosed plots was chosen to ensure they were situated in areas with different morphological characteristics. For example, plots A and C were placed in areas that had been previously cleared for fire prevention, plot D was located in a rocky area, and plot B was situated in an area with a higher initial amount of vegetation.

### Field sampling

A drone (DJI Phantom 4 PRO) was used to collect aboveground biomass data in the study area during four different periods of time (named “Time”, F1-4): September 2018, February 2019, December 2019, and November 2020. The collected data were used to evaluate the difference between the exterior and interior of the enclosed plots, to which the animals did not have access (Appendix A).

The biomass volume differential was calculated by reverse engineering techniques from point clouds corresponding to each plot and its surroundings. Images were standardized by removing noise from the point cloud, followed by calibration and geometric homogenization of the point cloud. The data clouds were thinned to 1 point/cm, upon which two work contours were established for each plot: one corresponding to the enclosed plot and its immediate surroundings and another referring to the 100-ha study area itself.

For each plot, two volumes were calculated: one corresponding to the biomass volume of the plot and its surroundings and another corresponding only to the enclosed plot itself.

A methodology has been developed to calculate the volume in both scenarios. It is based on iteratively computing the volume of prisms with a 5 cm x 5 cm tessellation and a height referenced to a horizontal theoretical comparison plane. This comparison plane is established at a minimum elevation value assigned to each plot, which is derived from the average elevation value of points on the vegetation surface cloud. The calculation involves weighting a minimum of 25 vegetation elevation points for each tile.

The horizontal plane of theoretical comparison chosen for the calculation of each volume was assigned for each plot and its surroundings according to its spatial layout, which was constant for the different times analysed. According to the comparison of the data and the differences observed between the data obtained, the volume differentials between both times were determined.

The slope was obtained for each plot and its surroundings using the 5 m resolution slope model from the Geographic National Center of Geographic Information of Spain [38]. Information about weather conditions during the survey days (relative humidity, wind speed and direction) was collected from the Galician government meteorological service [39].

### Statistical analysis

We performed all the statistical analyses in R [40]. We used the “firebehavioR” R package [41] to examine how the varying amounts of gorse biomass in the different plots would affect fire behaviour in shrub-dominated landscapes under various environmental and fuel-moisture conditions.

The effects of grazing on fire behaviour were modelled by using our shrub fuel estimates, calculated based on the biomass volumes collected via UAV, as input while holding parameters for other fuel and environmental conditions constant (for example, dead fuel loading, herbaceous fuel loading, and live and dead fuel moisture). The model settings used were developed based on inputs from several sources, including previously collected biomass data, ecological site descriptions, published literature, and existing fuel models in the “firebehavioR” package (see Appendix B for details on settings, parameters, and input).

There are several assumptions and caveats that should be considered when interpreting the results presented in this manuscript. The Rothermel Eqs. [42, 43], one of the three functions on which “firebehavioR” is based, assumes uniformity in fuel continuity, weather, and wind; no fire spotting (that is, fire starting from embers landing in advance of the fire front); no extreme fire behaviour; and surface fire only. These assumptions were not consistent since, for example, relative humidity changes from day to night, as does wind speed. However, these models provide a mechanism to compare the changes in fuel load that grazing and vegetation composition would most likely impact. The results provide fire behaviour predictions only for a free-running head fire at steady state. For simplicity, only results for the surface rate of spread (SROS) are presented because this fire behaviour variable is most easily understood and is indicative of overall fire behaviour.

We based our calculations on the shrub fuel model SH9: dense, finely branched shrubs with significant fine dead fuel, approximately 1 to 2 m tall, with the possible presence of herbaceous fuel. The parameters for the model – fuel load, surface-area-to-volume (SAV) ratio by component and size class, heat content by category, fuel bed depth and dead fuel moisture of extinction – were established as in Scott & Burgan [44]. This parameterization represents a very high-load, humid climate shrub. Models were run considering a high dead fuel moisture (DFM) of D4, based on the results of Anderson [45], and at varying live fuel moisture (LFM) levels, including 120 and 150%.

The effect of grazing on fuel hazard reduction was estimated by simulating potential wildfire behaviour using the Crown Fire Initiation and Spread (CFIS) model. Specifically, CFIS was used to estimate the probability of crown fire occurrence using a logistic model [46] and to classify fire into surface or crown fire differentiating passive from active crown fires as defined in Van Wagner [47].

The CFIS inputs used in the simulations included 10 m open wind speed (WS, km/h), estimated fine fuel moisture (EFFM, %), fuel strata gap (FSG, m), surface fuel consumption (SFC, kg/m^2^) and overstory bulk density (CBD, kg/m^3^) for each plot. The EFFM value (12%) was calculated using CFIS by selecting relative humidity states (RH: 58–80%) and constant temperature values (9–20 °C).

We quantified fuel loads (biomass) in terms of the vertical arrangement of the CBD, including combustible foliage and woody material, per unit volume. CBD is a useful metric for characterizing the structural effects of disturbances, including fires, and is also a key input in fire behaviour models such as “firebehavioR”. CBD has traditionally been measured using destructive methods but can also be modelled using nondestructive methods such as airborne lidar in this case. One particular strength of utilizing lidar-based CBD maps is the provision of detailed, spatially explicit information covering the entire study area.

Finally, we performed a linear mixed effects analysis using Restricted Maximum Likelihood (REML) estimation on the data using the “lme4” package [48]. The main goal of this analysis was to model the difference in the mean response between “grazing” and “non-grazing” areas, with SROS as our dependent variable, to determine if there was a statistically significant difference between areas with and without feral horses. The model was fitted specifying random effects of the enclosed plot ID (A-D), and the time of the measurement (F1-4).

## Results

The vegetation volume of the four plots was calculated in m^3^ (Table 1) based on the results from the UAV flights each time. The internal volume refers to the ungrazed plots and the external volume refers to the grazed areas immediately surrounding them.

The slope ranged from 3.4° (plot C) to 7.4° (plot D), with temperatures ranging from 9 °C in February 2019 to 20.8 °C in September 2018.

**Fig. 3:**
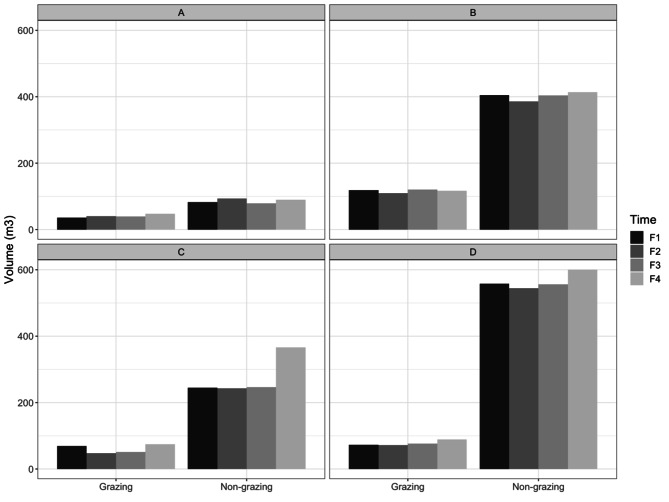
Volume differences (m^3^) for four enclosed plots and its surroundings measured with UAV from 2018 to 2020. Legend: Total volumes were measured during four time periods (F1 = September 2018, F2 = February 2019, F3 = December 2019 and F4 = November 2020) in four different plots (**A-D**). The non-grazing plots refer to the enclosed plots not accessible by Galician feral horses. Grazing plots are the immediate surroundings of the mentioned enclosed plots that were accessible by Galician feral horses

The aboveground volume differed between the grazed and ungrazed plots, averaging 74.0 ± 15.9 and 332.1 ± 97.7 m^3^ in the grazed and ungrazed treatments, respectively (Fig. 3). The volume increase was almost always lower in the grazed plots across the seasons than in their adjacent ungrazed plots, with averages of 2.6 ± 10.7 and 8.8 ± 44.9 m^3^, respectively.

**Table 1:** Internal, external and total volumes of biomass estimated using UAV measurements for the four plots

Time		Plot	Internal volume (m^3^)	External volume (m^3^)	Total volume (m^3^)
**F1**	September 2018	A	36.1	83.0	119.0
		B	118.5	405.0	523.5
		C	69.1	244.8	313.9
		D	73.2	631.4	558.2
**F2**	February 2019	A	39.0	93.7	133.5
		B	109.9	386.2	496.1
		C	47.4	243.3	290.7
		D	72.3	544.4	616.7
**F3**	December 2019	A	39.8	79.4	119.1
		B	120.6	403.8	524.5
		C	51.7	246.7	298.3
		D	76.8	556.5	633.3
**F4**	November 2020	A	47.3	89.6	136.8
		B	117.2	413.6	530.8
		C	74.2	366.0	440.2
		D	89.3	600.2	689.5

**Fig. 4:**
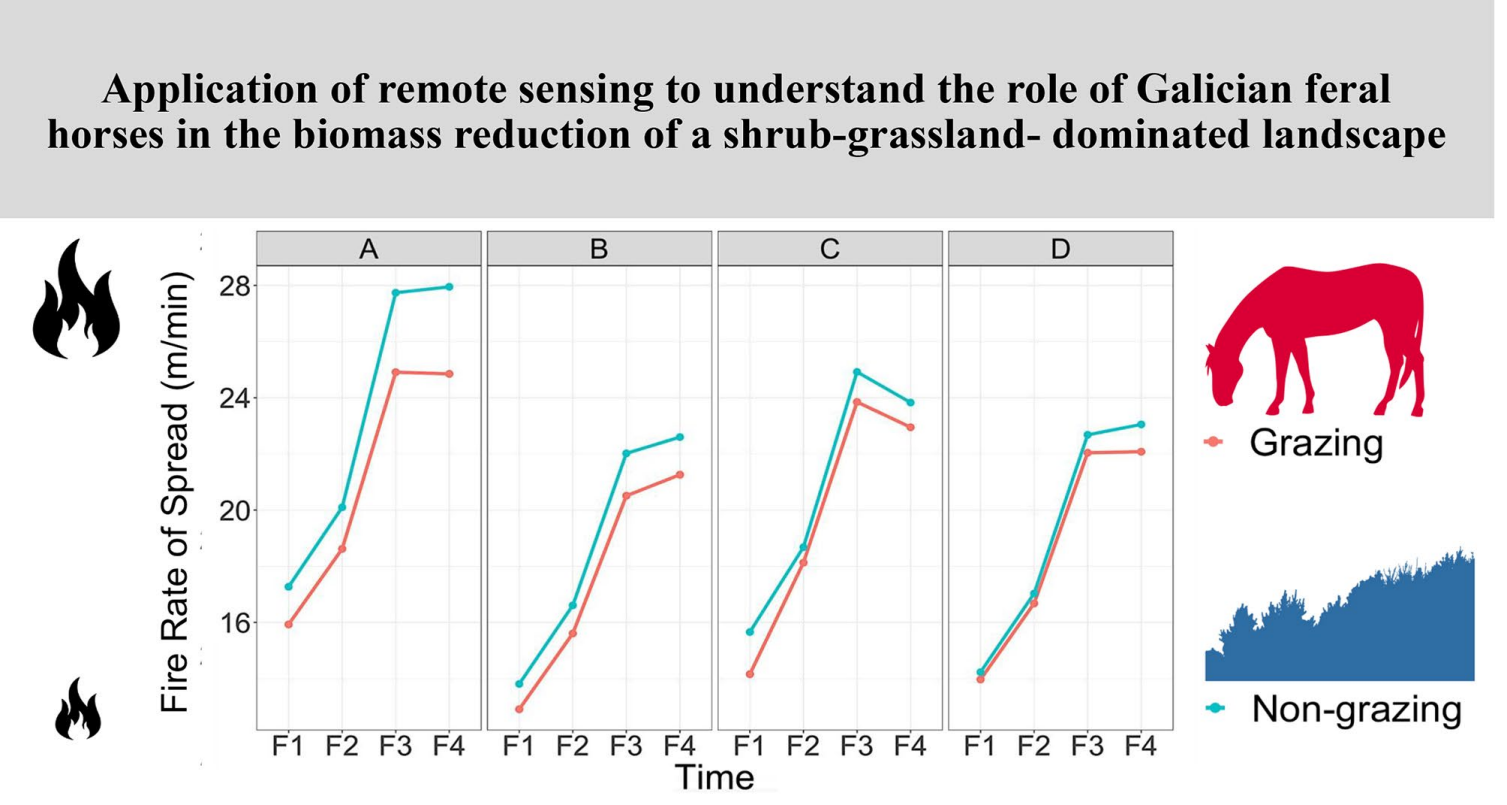
Fire Rate of Spread (m/min) estimates for the four plots from 2018 to 2020. Legend: Fire Rates of Spread were calculated for the four time periods where total volumes of biomass were collected (F1 = September 2018, F2 = February 2019, F3 = December 2019 and F4 = November 2020) in four different plots (**A-D**). The non-grazing plots refer to the enclosed plots not accessible by Galician feral horses. Grazing plots are the immediate surroundings of the mentioned enclosed plots that were accessible by Galician feral horses

The results of the fire-behaviour modelling system analysis identifying significant differences between wildfires on grazed and ungrazed plots are illustrated in Fig. 4. Reducing the levels of fuels, as accomplished by Galician feral horse grazing, reduced the modelled surface rate of spread in all four plots surroundings by a small but consistent amount.

In plot A, the SROS was between 1.34 (September 2018) and 3.10 (November 2020) m/min less where animals could graze. In plot B, the SROS varied between 0.90 (September 2018) and 1.07 (December 2019) m/min. The plot C values ranged between 0.55 and 1.50 m/min (February 2019 and November 2018, respectively). Plot D showed SROS levels ranging from 0.26 (November 2018) to 0.97 (November 2020) m/min in the grazing areas. The effects of reduced fuel load on fire behaviour were more pronounced at low wind speeds and high fuel moisture values. When burning conditions became extreme (more than 20 km/h wind speed), changes in the fuel load had little effect on the fire behaviour variables.

When analysing the results of the linear mixed effect model (Table 2), we can appreciate that the variance for the intercept associated with the random variable “Plot_ID” is 3.01, corresponding to a standard deviation of 1.73, indicating a moderate variability in SROS across the four different plots. The variance for the intercept associated with the variable “Time” is 19.47, with a standard deviation of 4.41, suggesting substantial variability in SROS over the four different time periods where the total volumes of biomass were collected.

**Table 2:** Outcome of the linear mixed effect model with SROS as response variable, “grazing” and “non-grazing” plots as fixed effects and the random effects of “time” (F1-4) and “plot ID” (A-D).

Random effects					
Groups name		Variance	Std. Dev		
Plot_ID (Intercept)		3.01	1.73		
Time (Intercept)		19.47	4.41		
Residual		0.39	0.63		
Number of obs: 32, groups: Plot, 4; Time, 4					
**Fixed effects**					
		**Estimate**	**Std. Error**	**t value**	**Pr(>|t|)**
(Intercept)		19.28	2.38	8.12	1.37 × 10^− 3^ **
Status Non-grazing		1.23	0.22	5.57	9.82 × 10^− 6^ ***
**Correlation of Fixed Effects**					
		**(Intr)**			
Status Non-grazing		-0.05			

The fixed effects estimate for the intercept (19.28) represents the estimated SROS mean in the grazed plots. The coefficient for the enclosed, ungrazed plots (1.23) indicates that the SROS mean is 1.23 m/min higher for the enclosed areas compared to its surroundings, where feral horses can graze freely. The standard error for this estimate (0.22), as well as the high t-value (5.57) and an extremely small p-value (9.82 × 10^-6^), suggests that this difference is statistically significant, implying that the presence of feral horses has a meaningful impact on SROS.

## Discussion

Our analysis of the effect of feral horse grazing on fuel load and hence the expected rate of spread of wildfire revealed a consistent decreasing effect. Although the exact benefit varies over time, it remains difficult to quantify exactly. However, grazing is preferable to nongrazing.

While grazing, Galician feral horses also have a moulding effect on gorse that prevents great heights and densities, as well as contributes to a reduction in the availability of fuel at the time of the possible threat of a fire, which highlights its ability to control biomass by reducing the chances of fires. In addition, it is probable that in the event of a fire, the effect of the Galician feral horse decreases the speed of advance and the virulence with which it affects the environment, a fact that is indicated as a line of future research of special interest. The rupture of the vegetal continuity allows better access to the means of extinction in the case of a fire, creating natural firebreakers.

The combination of the Galician feral horse grazing effect, together with correctly described burns, can reduce the vegetal load while it is renewed, resulting in the production of more palatable food. It is also worth noting the good coexistence observed between cattle and Galician feral horses, in addition to making good use of the resources that the environment offers by having different nutritional needs, since cattle are more herbivorous than lignivorous. This combination of loads adjusted to the territory can provide satisfactory results in terms of reducing plant biomass.

Galician feral horses not only limit the growth of gorse [49], thus reducing the risk of forest fires due to the gorse capacity to generate large quantities of highly flammable biomass [50] but also bring other environmental benefits. Their presence increases the richness and diversity of plant species, particularly rare species, characteristic of heathland communities and of significant conservation interest (e.g., *C. filipendulum, G. pneumonanthe, S. tinctoria*, and *S. humilis*) [51]. Other studies have also reported positive effects of horse grazing on floristic diversity in various plant communities, such as coastal and wet grasslands in France [52]. However, in more arid conditions, horse grazing can negatively impact plant diversity, as observed in rangelands in Nevada [53, 54].

Feral horses can take the edge off livestock attacks [55] and are also a representative feature of Galician tradition and heritage [56], by creating income from meat production, as well as bringing other benefits, such as landscape enhancement, improved access, and the production of secondary products such as mushrooms.

In these regards, sustainable land management is fundamental to containing wildfires [57–59]. More specifically, animal husbandry is considered an effective practice for the natural control of vegetation [60, 61]. The indirect control of traditional livestock such as sheep and goats, especially nomadic livestock or livestock managed in flocks, was demonstrated to be particularly effective in controlling fuel accumulation in forests, maquis/bushland, and nonforest natural land [62–65]. The economically sustainable use of prescribed grazing, as advocated by Taylor [66] and Diamond et al. [67], encompasses various applications, such as maintenance grazing of fuel breaks with mixed horse-cattle herds, high impact browsing in areas where prescribed burns are impractical due to its high cost, and follow-up management in burned areas (as a short-term strategy). Feral horses have emerged as the most cost-effective, nontoxic, and nonpolluting solution available. They are highly valued by the general public and offer an environmentally friendly and effective approach for nearly carbon-neutral fuel control [68], deserving further attention and applied research. However, the presence of traditional husbandry may not be possible in some areas, for instance, remote locations with no human population or where horses cannot survive due to a lack of appropriate food or extreme weather and geographical conditions.

Assuming that livestock grazing with wild animals is an effective tool for mitigating fire risk [69, 70], the continuous decrease in livestock density, especially in nomadic flocks, because of multiple drivers associated with urban growth and agricultural decline in fringe districts, as observed in the Galicia region, is an important factor shaping fire risk in peri-urban areas. In such contexts, moderate grazing was already recognized to reduce fire severity in previous studies, positively influencing fuel characteristics [71–74]. More specifically, a previous study in the same area [49] revealed a significant reduction and transformation of plant biomass, more specifically gorse by horses. In that study, an average intake per horse of 18.5 kg of green matter ingested per day was established, which is a total of 6,753 kg/horse. More than half of that weight (3,581 kg year/horse) corresponds exclusively to the consumption of gorse.

Using UAVs for collecting data on gorse biomass volumes provided a fast and precise way to gather high-resolution spatial information without disturbing local wildlife populations and their habitat. The aerial surveys allowed us to cover the study area comprehensively and quickly, enabling faster data collection and analysis compared to traditional methods [75]. Our research demonstrates that feral horse grazing is a preemptive treatment that can alter fire behaviour, burned area and fire intensity in at least some wildfires in areas dominated by gorses in the northwestern Iberian Peninsula and likely other shrub grasslands. Obviously, the effects of grazing may be moderated in fires that occur under more extreme weather conditions and in plant communities with greater amounts of woody vegetation. Nonetheless, feral horse grazing can be applied across vast rangeland landscapes where other fuel management treatments are too expensive or impractical to apply or where traditional grazing (sheep, goats, etc.) is not available. Further refinement and evaluation across a variety of plant community types and in different areas with varying geographies and fire weather conditions would be of great additional value to our findings.

### Electronic supplementary material

Below is the link to the electronic supplementary material.

Supplementary Material 1

Supplementary Material 2

## Data Availability

Datasets generated during the current study are available from the corresponding author on reasonable request. The UAV LiDAR data has been provided by Xeométrica (https://www.xeometrica.com/) but restrictions may apply to the availability of these data, which were used under license for the current study, and so are not publicly available.
