# Supplementary material for: Application of remote sensing to understand the role of Galician feral horses in the biomass reduction of a shrub-grassland-dominated landscape
Source: BMC Ecol Evol. 2024 Jul 2;24:89. doi: 10.1186/s12862-024-02276-5 (PMC11218180; doi:10.1186/s12862-024-02276-5)
Supplement: Supplementary file 1 — Supplementary Material 1 [file 12862_2024_2276_MOESM1_ESM.docx]

**Appendix A** - Refined 3D point cloud used for volume calculation in each enclosed plot and its surroundings. Flights were done four times between September 2018 and November 2020.

| **Time** | | **Enclosed plot** | **Refined 3D point cloud for total volume**  **(Millions of points)** | **Refined 3D point cloud for plot volume**  **(Millions of points)** | **Total flat surface including surroundings**  **(m^2^)** | **Enclosed plot flat surface**  **(m^2^)** | **Elevation**  **(m)** |
| --- | --- | --- | --- | --- | --- | --- | --- |
| F1 | September 2018 | A | 1.4M | 0.5M | 190.9 | 54.3 | + 786.0 |
|  |  | B | 4.4M | 1.0M | 287.9 | 59.2 | + 787.5 |
|  |  | C | 2.8M | 0.7M | 337.3 | 56.1 | + 786.4 |
|  |  | D | 4.6M | 0.5M | 464.0 | 55.1 | + 788.0 |
| F2 | February 2019 | A | 1.4M | 0.4M | 190.9 | 54.3 | + 786.0 |
|  |  | B | 4.3M | 0.9M | 287.9 | 59.2 | + 787.5 |
|  |  | C | 1.8M | 0.3M | 337.3 | 56.1 | + 786.4 |
|  |  | D | 3.6M | 0.4M | 464.0 | 55.1 | + 788.0 |
| F3 | December 2019 | A | 0.4M | 0.1M | 190.9 | 54.3 | + 786.0 |
|  |  | B | 1.3M | 0.3M | 287.9 | 59.2 | + 787.5 |
|  |  | C | 1.2M | 0.2M | 337.3 | 56.1 | + 786.4 |
|  |  | D | 10.8M | 1.2M | 464.0 | 55.1 | + 788.0 |
| F4 | November 2020 | A | 0.6M | 0.2M | 190.9 | 54.3 | + 786.0 |
|  |  | B | 1.1M | 0.2M | 287.9 | 59.2 | + 787.5 |
|  |  | C | 2.1M | 0.3M | 337.3 | 56.1 | + 786.4 |
|  |  | D | 6.0M | 0.7M | 464.0 | 55.1 | + 788.0 |
