# Supplementary material for: Application of remote sensing to understand the role of Galician feral horses in the biomass reduction of a shrub-grassland-dominated landscape
Source: BMC Ecol Evol. 2024 Jul 2;24:89. doi: 10.1186/s12862-024-02276-5 (PMC11218180; doi:10.1186/s12862-024-02276-5)
Supplement: Supplementary file 2 — Supplementary Material 2 [file 12862_2024_2276_MOESM2_ESM.docx]

**APPENDIX B**

Fire behavior model settings, parameters and input used with FirebehavoR R package.

**Fuel Model SH9 parameters:**

Description: Very high load, humid climate shrub

Source: Scott & Burgan (2005)

| **Fuel load (t/ac)** | | | | | **Fuel model type^a^** | **SAV ratio (1/ft)^b^** | | | **Fuel bed depth (ft)** | **Dead fuel extinction moisture (%)** | **Heat content (BTU/lb)^c^** |
| --- | --- | --- | --- | --- | --- | --- | --- | --- | --- | --- | --- |
| **1-hr** | **10-hr** | **100-hr** | **Live herb** | **Live woody** |  | **Dead  1-hr** | **Live herb** | **Live woody** |  |  |  |
| 4.50 | 2.45 | 0.00 | 1.55 | 7.00 | dynamic | 750 | 1800 | 1500 | 4.4 | 40 | 8000 |

a Fuel model type does not apply to fuel models without live herbaceous load.
b Surface-area-to-volume.
c The same heat content value was applied to both live and dead fuel categories.

**Fuel Moisture D4L1 parameters:**

Description: Very high dead fuel moisture content, fully green (uncured) live fuel moisture content.

Source: Scott & Burgan (2005)

|  | **D4**  **High (%)** |  |  | **L4**  **Fully green (uncured)**  **High (%)** |
| --- | --- | --- | --- | --- |
| 1-hr | 12 |  | Live herbaceous | 120 |
| 10-hr | 13 |  | Live woody | 150 |
| 100-hr | 14 |  |  |  |

**Canopy Fire Initiation and Spread (CFIS) model settings and input:**

Description: Crown fire rate of spread in m/min.

Source: Alexander and Cruz (2006)

cfis(fsg, u10, effm, sfc, cbd, id)

| fsg | fuel stratum gaps (m). Equivalent to canopy-base height (CBH) |
| --- | --- |
| u10 | 10-m open wind speeds (km/hr). Data obtained from the Galician government meteorological service (Meteogalicia, 2022) |
| effm | effective fine fuel moistures (%). Based on Fuel Moisture model D4 |
| sfc | surface fuel consumed (Mg/ha). Based on Fuel model SH9 |
| cbd | canopy bulk densities (kg/m3). Obtained using UAV |
| id | spot ignition delays, the time during which a given firebrand generates, is transported aloft, and ignites a receptive fuelbed (min). Assumed the default 1 as per Alexander and Cruz (2006) |
